# Spatial Cluster Detection of Air Pollution Exposure Inequities across the United States

**DOI:** 10.1371/journal.pone.0091917

**Published:** 2014-03-19

**Authors:** Bin Zou, Fen Peng, Neng Wan, Keita Mamady, Gaines J. Wilson

**Affiliations:** 1 School of Geosciences and Info-Physics, Central South University, Changsha, Hunan, China; 2 Department of Geography, University of Utah, Salt Lake City, Utah, United States of America; 3 Department of Epidemiology and Health Statistics, School of Public Health, Central South University, Changsha, Hunan, China; 4 Department of Biological Sciences, Huston-Tillotson University, Austin, Texas, United States of America; Stony Brook University, Graduate Program in Public Health, United States of America

## Abstract

Air quality is known to be a key factor in affecting the wellbeing and quality of life of the general populous and there is a large body of knowledge indicating that certain underrepresented groups may be overexposed to air pollution. Therefore, a more precise understanding of air pollution exposure as a driving cause of health disparities between and among ethnic and racial groups is necessary. Utilizing 52,613 urban census tracts across the United States, this study investigates age, racial, educational attainment and income differences in exposure to benzene pollution in 1999 as a case. The study examines spatial clustering patterns of these inequities using logistic regression modeling and spatial autocorrelation methods such as the Global Moran's *I* index and the Anselin Local Moran's *I* index. Results show that the age groups of 0 to 14 and those over 60 years old, individuals with less than 12 years of education, racial minorities including Blacks, American Indians, Asians, some other races, and those with low income were exposed to higher levels of benzene pollution in some census tracts. Clustering analyses stratified by age, education, and race revealed a clear case of disparities in spatial distribution of exposure to benzene pollution across the entire United States. For example, people aged less than 4 years from the western south and the Pacific coastal areas exhibit statistically significant clusters. The findings confirmed that there are geographical-location based disproportionate pattern of exposures to benzene air pollution by various socio-demographic factors across the United States and this type of disproportionate exposure pattern can be effectively detected by a spatial autocorrelation based cluster analysis method. It is suggested that there is a clear and present need for programs and services that will reduce inequities and ultimately improve environmental conditions for all underrepresented groups in the United States.

## Introduction

Environmental injustice may be defined as a type of injustice when a particular social group is disproportionately burdened with environmental hazards [1]. The underlying contributors to environmental injustices can be political, economic, historical, and social [2].

Air pollution, the most common type of pollutant in environmental injustice studies, can be traced back to the industrialization-urbanization nexus beginning in the 19^th^ century. Evidence indicates that air pollution exposure is more serious than previously thought, in terms of adverse health impacts such as reduced life expectancy, increased daily mortality and hospital admissions, birth outcomes, and asthma [3]. These effects have been shown to exist in both economically developing and developed countries [4]. Systematic efforts to control air pollution and to protect public health commenced mostly in the second half of the 20th century and have intensified since the 1960s [5].

Exposure to air pollution, however, may vary spatially within a city [6] and these variations may follow social gradients that influence susceptibility to environmental exposures [7]. Residents of poorer neighborhoods may live closer to point sources of industrial pollution or roadways with higher traffic density [8]. International research has shown that air pollution exposure varies by socio-economic status, with lower socio-economic groups being disproportionately exposed to air pollution and to environmental mechanisms that lead to inequities in health [9]. For example, there is consistent evidence in California that patterns of disproportionate exposure to air pollution among minority and lower-income communities exists [10]. These communities also face other challenges associated with low socioeconomic status, including psychosocial stressors, which make it more difficult to cope with these exposures [9].

Meanwhile, although current research has confirmed the relations between social-demographic characteristics (e.g., education, age, race etc.) and disease [11], they are still inadequate in explaining the underlying reasons for disease disparities. Thus, further understanding of the role of socio-demographic status as a component of susceptibility to the adverse health effects of air pollution is necessary in the process of setting ambient air quality standards and implementing programs and policy that lead to adherence to these standards.

Today, air pollution is still a major environmental health issue in the United States, directly affecting people's wellbeing and quality of life with adverse health impacts such as excess respiratory, cardiovascular morbidity and higher mortality [12]. International survey data showed a 7–10% premature birth rate in industrialized countries, and specifically 9–12% in United States in recent years, with the trend for both showing an increase [13]. In this way, a broader understanding of the causes of population health disparities by race/ethnicity, socioeconomic status, and geographic location is necessary for achieving better solutions to population health problems caused by the complex cocktail of air pollution found in the United States. This study aims to investigate census tract level exposure to air pollution by these factors and to examine the spatial clustering patterns of the disparities at county level.

## Data and Methods

### Study Area

**Figure 1:**
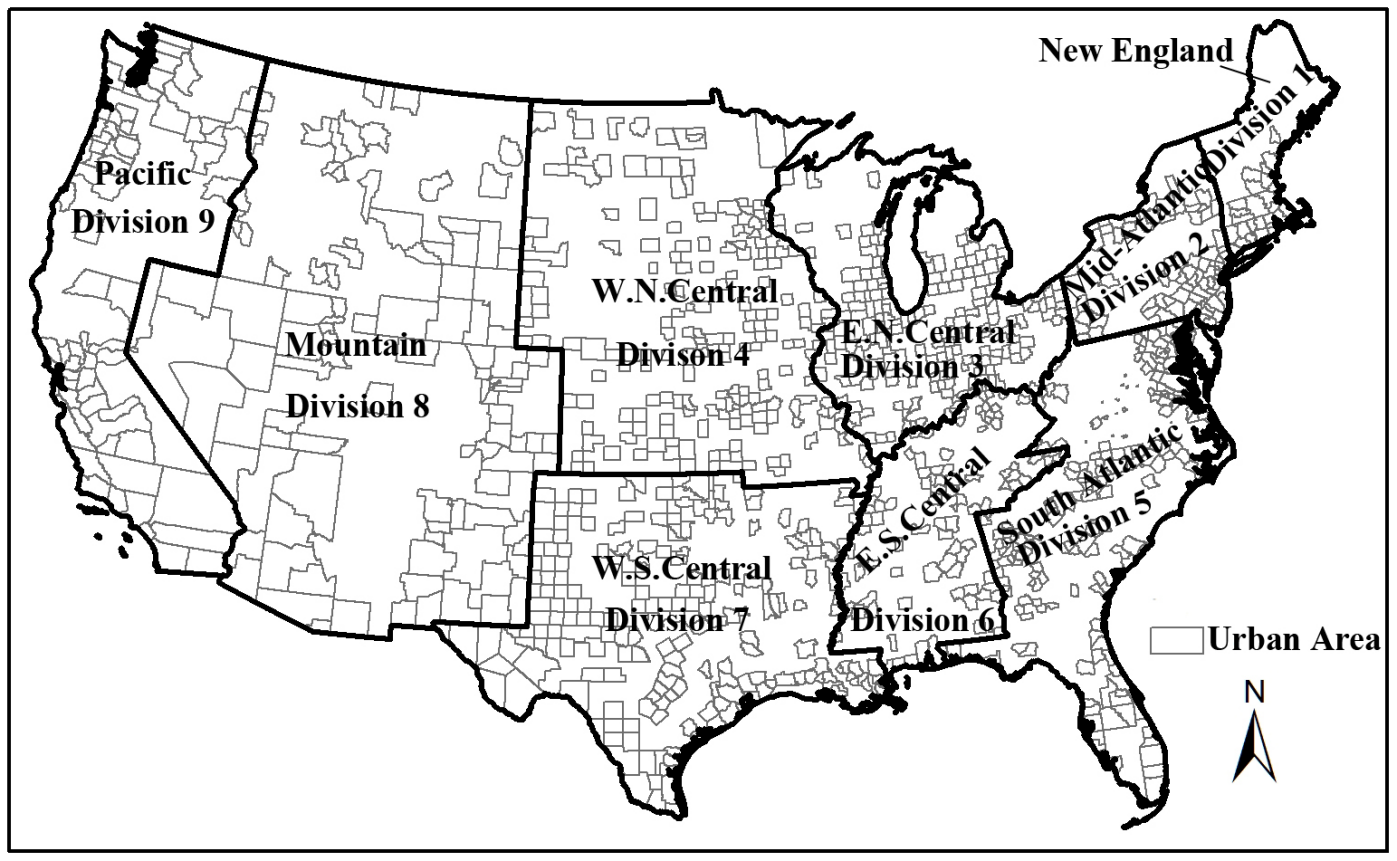
United States census divisions of urban-designated counties containing 52, 613 tracts used in this study. The study area focuses on all urban census tracts within the United States, which is further classified into nine divisions. It consists of an aggregate number of 52,613 census tracts within 48 contiguous states and Washington DC.

This study focuses on all urban census tracts within the United States, which is further classified into four census regions (e.g. Northeast, Midwest, West, South), and nine divisions [14]. This regional and divisional classification, as defined by the United States Census Bureau, is based upon factors such as employment, crime, health, consumer expenditures, and housing. The demographic differences between these divisions are suitable to be utilized for analyzing the air pollution exposure inequities across the entire country. We chose to use census tracts because this was the smallest level of aggregation at which air quality information for benzene was available and it was generally utilized as the standard spatial scale for environmental justice studies due to its relatively homogeneous characteristics relative to socio-demographic status and living conditions [15], [16]. The study area consists of an aggregate number of 64,890 census tracts, 3,109 counties within 48 contiguous states and Washington DC. The number of counties included in our study is 29 for New England (Division 1), 81 for Mid-Atlantic (Division 2), 174 for East North Central (Division 3), 187 for West North Central (Division 4), 214 for South Atlantic (Division 5), 97 for East South Central (Division 6), 194 for West South Central (Division 7), 101 for Mountain (Division 8), and 69 for Pacific (Division 9) (Fig. 1). After filtering out rural census tracks, we were left with 52,613 urban census tracts that account for 80.5% of the total 64,890 census tracts in the United States.

### Data Sources and Analysis

The Environmental Hazard Data were ascertained from the US Environmental Protection Agency's (EPA) NATA (National Air Toxics Assessments) website [17]. The NATA data is the EPA's ongoing comprehensive evaluation of air toxics in the U.S. EPA developed the NATA as a state-of-the-science screening tool for state, local, and tribal agencies to prioritize pollutants, emission sources, and locations of interest and for researchers to gain a better understanding of environmental risks. These datasets are particularly suitable for environmental justice research, not only because they allow researchers to estimate the potential health risks associated with specific environmental hazards and analytical spatial units, but also because the data modeling takes into account a number of factors such as wind speed, wind direction, air turbulence, smokestack height and the rate of chemical decay and deposition [18]. Another important advantage of the NATA data is their spatial compatibility with socio-demographic census data: the modeled risk estimates are available for census units (e.g., tracts), which also include demographic characteristics of residential population.

**Figure 2:**
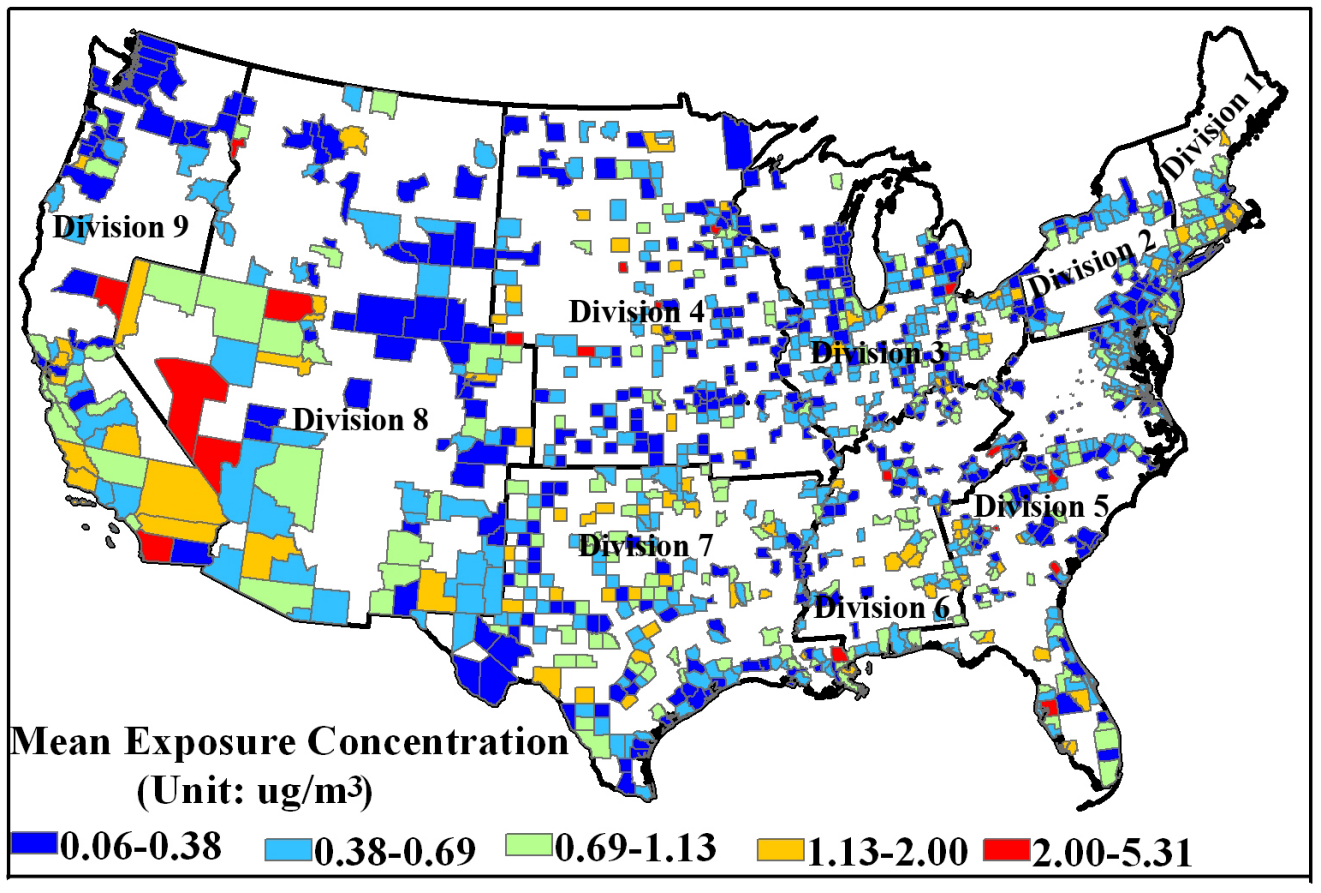
Annual exposure concentrations of total benzene at the census tract level in the United States. Annual exposure concentrations of census tracts have been utilized to calculate county level mean exposure concentration values, which was used as a ‘relative exposure level’ metric to evaluate benzene pollution exposure inequities. Division 1 is New England; Division 2 is Mid-Atlantic; Division 3 is East North Central; Division 4 is West North Central; Division 5 is South Atlantic; Division 6 is East South Central; Division 7 is West South Central; Division 8 is Mountain; Division 9 is Pacific.

The annual benzene pollution concentration for census tracts was used to represent air pollution. Benzene is a ubiquitous chemical in the environment that causes acute leukemia and probably other hematological cancers [19]. Meanwhile, recent studies reported an association between higher benzene exposure concentrations with lower social economy status and social class [20], [21]. While other air pollutants (e.g. sulfur dioxide) have experienced a downward trend in use over the past few decades, benzene is still one of the key toxic air pollutants produced by today's petrochemical industry and can be found in gasoline petroleum tanks throughout urban areas. Benzene exposure data from 1999 NATA have been utilized for air pollution exposure equity analysis [22], [23]. We calculated county level mean exposure concentration values based on exposure concentrations of census tracts (Fig. 2). Because recent studies have focused on the effects of continuous exposure to low concentrations of benzene [24], [25], [26], we used a ‘relative exposure level’ metric to evaluate benzene pollution exposure inequities in this study [27]. In this way, population in census tracts with exposure concentrations higher than a county level mean exposure concentration value are recognized as ‘high’ exposure concentration, whereas as low exposure concentration is assigned to census tracts below average.

**Figure 3:**
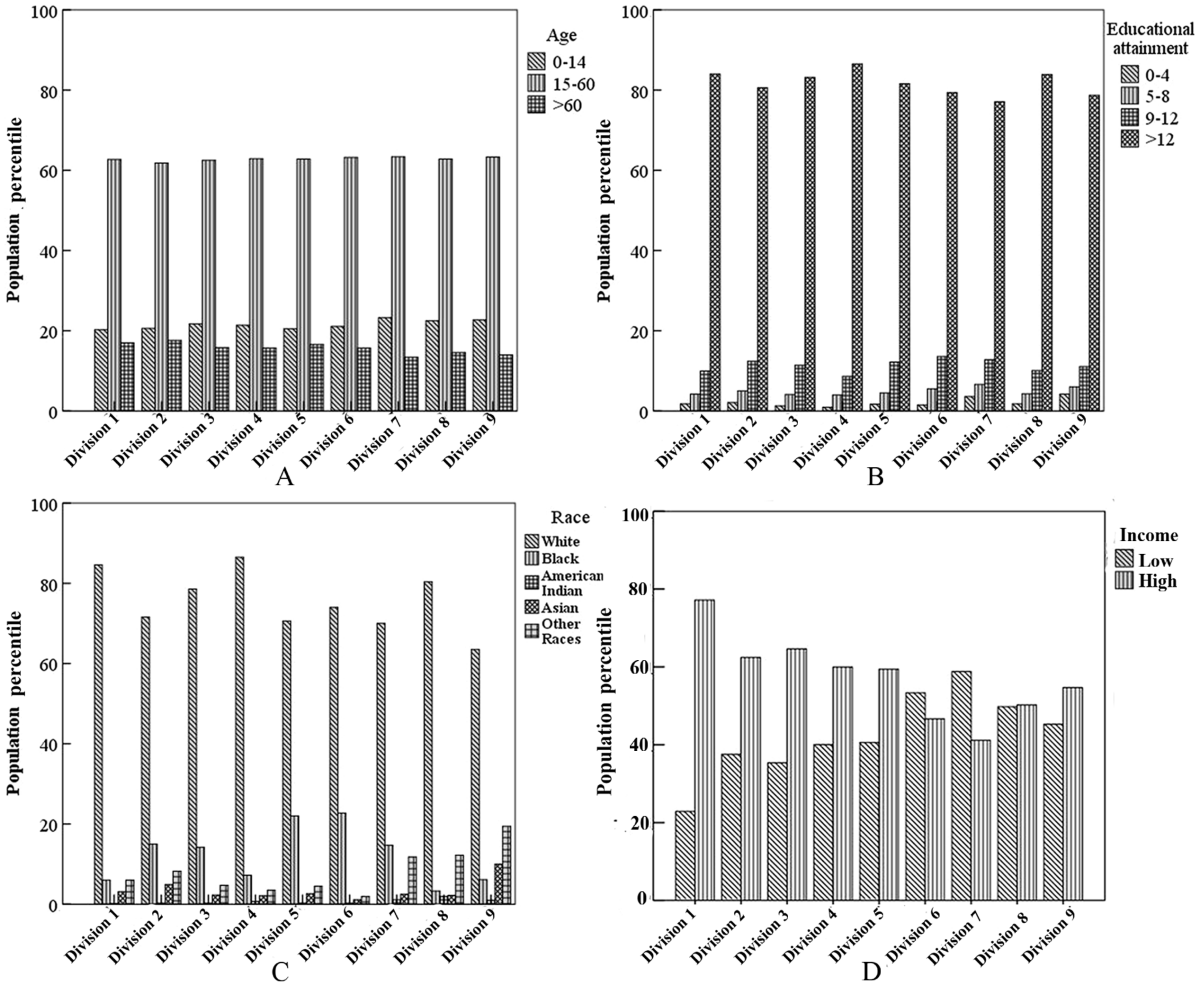
Population percentiles based on socio-demographic characteristics in the nine divisions. (A): Population percentile based on age characteristic in the nine divisions. Age group 15 to 60 have the highest population percentage. (B): Population percentile pertaining to educational attainment characteristic in the nine divisions. Educational attainment more than 12 years have the highest population percentage. (C): Population percentile of race characteristic in the nine divisions, the white have the highest population percentage. (D): Population percentile of income characteristic in the nine divisions. Division 1 is New England; Division 2 is Mid-Atlantic; Division 3 is East North Central; Division 4 is West North Central; Division 5 is South Atlantic; Division 6 is East South Central; Division 7 is West South Central; Division 8 is Mountain; Division 9 is Pacific.

The population data at census tract and county levels in this study were retrieved from the US Census 2000 Summary File 1 [28], while the geographic boundaries of spatial scale were acquired from the Census 2000 Topologically Integrated Geographic Encoding and Referencing (TIGER)/Line dataset [29]. Following previous studies [30], [31], [32], [33], we selected age, race, educational attainment, and income as the socio-demographic indicators in this study. These characteristics were categorized into different levels based on the reference categories of existing studies [34] (Table 1). We reclassified the census tract level individual incomes into high or low levels (groups) by using the computed national wide median income values as standards. Population in census tracts with income values higher than the nation-wide median income value were categorized in the ‘high’ income group, whereas the ‘low income’ group was assigned to census tracts below that national average. Figure 3 shows the population percentiles based on socio-demographic characteristics in the nine divisions. It can be seen that the socio-demographic characteristics including age, race, education attainment, and income fluctuate significantly across the nine divisions. This again emphasizes the necessity of conducting demography-based analysis of air pollution exposure inequities.

**Table 1:** Standards of categorization and reference categories for socio-demographic measurements.

Characteristics	Level 1	Level 2	Level 3	Level 4	Level 5
Age	0–14	14–60*	>60	—	—
Race	White*	black	American Indian	Asian	Other races
Education attainment (years)	0–4	5–8	9–12	>12*	—
Income (US$)	<19000	> = 19000**	—	—	—

*Reference category for comparison based on existing studies in the environmental justice literature.

**The classification standard for income is detailed in the text.

### Spatial Cluster Analysis

Spatial autocorrelation is an optimal method for systematically ascertaining spatial patterns of air pollution exposure inequities [35]. For the purpose of detecting spatial clusters of environmental inequity across the United States, the spatial cluster analytical strategy used in this study is designed to include three sub-processes, including global autocorrelation analysis, logistic regression modeling, and local hot spot detection. Since we are interested in spatial patterns based on a large data set in the study area, it is reasonable that spatial dependence exists at the global scale because of the continuous characteristic of terrain in developed or open areas. Global autocorrelation analysis is therefore adopted to preliminarily explore the spatial autocorrelations of benzene pollution concentration as well as socio-demographic indicators. Odds ratios (*OR*s) were calculated for each county across the entire study area to further diagnose whether the environmental inequities were caused by the interactions among these different global scale spatial autocorrelations. Logistic regression modeling was used to calculate the ORs. Finally, local hotpot detection was employed to pinpoint the statistically significant hot spots or cluster areas based on the *OR*s of counties. The methodological principles and implementation details of these sub-processes are described as follows:

#### 1. Global autocorrelation analysis

At present, there are many ways to test the global autocorrelations of events. The most popular one among them is Moran's *I* statistic, which has been used to test the null hypothesis that the spatial autocorrelation of a variable is zero [36], [37]. If the null hypothesis is rejected, the variable would be considered spatially autocorrelated. Moran's *I* statistic of spatial autocorrelation is presented by Cliff and Ord 1981 as formulas (1–2) [38]:
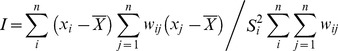
(1)

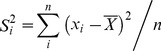
(2)where the global Moran's *I* index indicates the extent of global spatial autocorrelation of a variable, with the value ranging from −1.0 to +1.0, *n* denotes the number of all spatial units, *x_i_* and *x_j_* are the attribute values of a variable at spatial unit *i* and *j*, respectively, 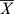 is the mean of attribute values of *x*, *S_i_* is the deviation of an attribute value at spatial unit *i* from its mean 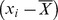, *w* denotes the space matrix, and *w_ij_* represents the spatial weight between spatial unit *i* and *j*.

In this study, we use the census tract as the base spatial unit. Moran's *I* index means the extent of global spatial autocorrelations of benzene pollution concentration as well as socio-demographic indicators (i.e. age, race, educational attainment, and income). The variable *x* in formulas (1) and (2) is therefore the attribute value of either ‘benzene pollution concentration’ or ‘a socio-demographic indicator’ such as ‘age’. *w_ij_* is determined based on the adjacency standard. Agency standard is when a shared side occurs between two adjacent census tracts *i* and *j*, then *w_ij_*  =  1, otherwise *w_ij_*  =  0. In order to verify the necessity of detecting local spatial clusters of potential environmental inequities, the global autocorrelation analyses in this study were implemented for entire United States and each Division separately. The analyses were conducted using the ‘Spatial Statistic Tools’ in ArcGIS 10.0.

#### 2. Logistic regression modeling

Logistic regression is a mathematical modeling technique that describes the relationship between several independent variables and a dichotomous dependent variable [39]. Most environmental justice studies use logistic regression to derive *OR*s based on the following formulas (3–8):
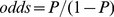
(3)

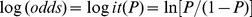
(4)

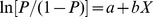
(5)

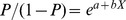
(6)

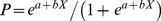
(7)

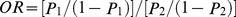
(8)where ‘odds’ is the probability of the dichotomous dependent variable equals an event (i.e. the case or control group being exposed to air pollution) (i.e., ‘*p*’) divided by the probability of the event not to occur (i.e., ‘*p/1-p*’). *OR* denotes odd ratio, indicating the relative value by which the ‘odds’ of the outcome increases (i.e., OR greater than 1.0) or decreases (i.e., OR less than 1.0). ‘*e*’ is the exponential constant, equal to 2.71828. ‘*P_1_*’ denotes the probability of the case group being exposed to air pollution. ‘*P_2_*’ denotes the probability of the control or reference group being exposed to air pollution. ‘*X’* represents the explanatory variables which are either interval-level or ‘dummy’, *a*, *b* represents partial regression coefficients of the independent variable ‘*X’*.

The logistic regression modeling in this study was implemented in SPSS version 17. In this process, the census tract level benzene pollution concentration was dichotomized as the dependent variable and coded as either ‘1’ (i.e. above) or ‘0’ (i.e. below) based on the mean concentrations at the county level. Consequently, age, race, educational attainment, and income were selected as independent variables and recoded (e.g., the reference category was coded as ‘0’). Meanwhile, the population amount of above/below pollution concentrations in each category by different socio-demographic indicators were input correspondingly as weight cases while the ‘indicator option’ in SPSS was set first as the reference category. In addition, we assess whether there is any significant relationship between the dependent variable *Y* (i.e. benzene pollution concentration) and independent variables *X* (socio-demographic indicators). More specifically, if any of the null hypotheses that *b* = 0 is valid, then *X* is statistically insignificant in the logistic regression model. However, it was difficult for us to eliminate the potential bias of the logistic regression modeling for each type of demographic variable (e.g., age) by inputting the remaining variables (e.g., race, education attainment, income) as confounding factors, because the attribute values for those variables were aggregated values rather than individual level ones.

#### 3. Local hot spot detection

When underlying global autocorrelation is detected, the question about how to identify more local patterns emerges. This leads to the challenge of finding an appropriate test for local spatial autocorrelations in the presence of global spatial autocorrelation. Local Moran's *I* based cluster mapping has been suggested as an effective method in detecting the hot spots or cluster areas of environmental exposure inequity based on spatial autocorrelation theory [40]. Formulas (9–11) present the basic principle of local Moran's *I* statistic. 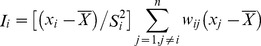
(9)

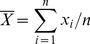
(10)

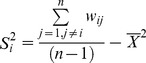
(11)where the designations for the letters such as *n*, *x_i_*, *x_j_*, 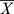
are similar to those in formulas 3–8, *S_i_* is the deviation between an attribute value at spatial unit *i* and its mean 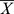, *I_i_* is the Moran's *I* index which indicates the extent to which neighboring spatial units congregate with each other in terms of attributes. If the attribute values in the dataset tend to cluster spatially (i.e., high values near high values; low values near low values), the Moran's *I* index will be positive. When high values repel other high values, or tend to be near low values, the index value will be negative. If the values in the dataset tend to scatter spatially, the index will be near zero. The range of the index value falls between −1.0 and +1.0 [40].

We utilized cluster and outlier analysis (Anselin Local Moran's *I*) functions in ‘Spatial Statistic Tools’ within ArcGIS 10.0 to identify the local hot spots or cluster areas of benzene exposure inequity in this study. In this process, *x* is the *OR* value of each county. *w_ij_* is determined based on the adjacency standard, where *w_ij_*  =  1 when there is a shared side between adjacent two counties, and 0 otherwise. The significance of the hot spots or cluster areas is determined by the Z-score and *P* value. That is, a high positive Z-score for benzene exposure inequities of a county with *P* value at 0.05 level indicates the surrounding features have the either high or low *OR* values (i.e., High-high, or Low-low). Inversely, a low negative Z-score for benzene exposure inequities of a county with *P* value at 0.05 level indicates a significant spatial outlier (i.e., High-low, or Low-high).

## Results

### Global Autocorrelation Analysis of Air Pollution Exposure

Table 2 delineates the values derived from the Global autocorrelation calculation for the nine divisions and the entire United States. With Global Moran's *I* index, people aged less than 4 years from Divisions 7 (0.090) and 9 (0.110) exhibit statistically significant clusters and have larger positive index values than the entire United States (0.046). Furthermore, even though Global Moran's *I* index values for Asians with educational attainment of 9-12 years appeared not to be statistically significant for the United States, Asians for Divisions 2, 4 and 5 (0.120, 0.054, 0.044), and education level of 9-12 years for Divisions 7 and 9 (0.058, 0.195) show significant cluster patterns.

**Table 2:** Global Moran's I statistic values by age, race, and educational attainment in the United States and the nine divisions.

	Age		Race				Educational attainment			Income
	<14	>60	Black	American Indian	Asian	Other races	<4	5–8	9–12	Low income
United States	0.046*	0.021*	0.033*	0.031*	0.003	0.028*	0.036*	0.021*	0.011	0.022*
Division 1	−0.071	−0.062	−0.105	−0.060	−0.061	−0.091	−0.065	0.083	−0.057	−0.007
Division 2	−0.118	0.084*	0.070*	−0.073	0.120*	−0.003	−0.019	0.213*	0.035	−0.069
Division 3	0.012	0.042	0.041*	−0.004	0.016	−0.002	0.026	−0.022	−0.018	−0.063
Division 4	−0.019	−0.010	0.032	0.025	0.054*	0.010	−0.005	0.050	0.007	0.051
Division 5	0.010	−0.022	−0.006	0.035*	0.044*	0.031	0.025	−0.022	−0.034	0.0004
Division 6	−0.007	0.159*	0.024	−0.175*	−0.005	−0.048	−0.016	0.090	0.088	0.033
Division 7	0.090*	0.050*	0.008	0.021	0.020	0.104*	0.026	0.114*	0.058*	−0.108
Division 8	−0.020	−0.019	0.009	0.053	0.004	−0.060	0.034	0.027	−0.000	−0.114
Division 9	0.110*	−0.027	0.051	0.226*	−0.015	0.088	−0.016	0.078	0.195*	−0.073

* p<0.05.

### Spatial Clustering of Air Pollution Exposure Inequity by Age

Table 3 delineates frequency of *OR*s greater than 1 by age characteristic at the county level in the United States and by the nine divisions. From Table 3, it can be seen that people belonging to age groups 0 to 14 and 60+ years old were exposed to higher levels of benzene pollution in some counties across the United States. For the age group of 60 years and older, Division 6 had the highest proportion (58.8%), followed by Division 2 (54.3%) and Division 7 (47.4%). The smallest proportion for that age group was found in Division 9 (34.8%). For the age group of 0–14, Division 9 displayed the greatest exposure (43.5%) followed by Divisions 7 and 1 (40.7%; 31.0% respectively). Division 8 has the lowest exposure in that age group (10.9%). We also observed that the proportion of counties exposed to higher levels of benzene pollution by division is mostly less than 50% for the United States and the nine divisions, except for the age group of 60 years and older in Divisions 2 and 6.

**Table 3:** Frequency of *OR*s greater than 1 by age characteristic at the county level in the United States and the nine divisions.

	Age	Count (percentage)	Minimum (95%CI)	Maximum (95%CI)	Mean
United States	0–14	306(26.7%)	1.017(1.006,1.029)	5.911(4.935,7.081)	1.189
	>60	537(46.9%)	1.010(1.001,1.019)	5.400(4.856,6.005)	1.285
Division 1	0–14	10(31.0%)	1.018(1.005,1.032)	1.322(1.286,1.359)	1.131
	>60	11(37.9%)	1.027(1.004,1.050)	1.451(1.415,1.488)	1.184
Division 2	0–14	10(12.3%)	1.028(1.008,1.049)	1.180(1.125,1.237)	1.098
	>60	44(54.3%)	1.010(1.001,1.019)	1.407(1.383,1.431)	1.183
Division 3	0–14	52(29.9%)	1.020(1.005,1.034)	1.472(1.404,1.543)	1.106
	>60	84(48.3%)	1.030(1.025,1.035)	1.730(1.505,1.990)	1.220
Division 4	0–14	22(11.8%)	1.043(1.002,1.085)	1.302(1.154,1.470)	1.135
	>60	82(43.9%)	1.055(1.009,1.104)	2.243(2.142,2.348)	1.301
Division 5	0–14	66(30.8%)	1.017(1.001,1.032)	5.026(4.502,5.610)	1.209
	>60	96(44.9%)	1.029(1.016,1.042)	5.400(4.856,6.005)	1.333
Division 6	0–14	27(27.8%)	1.028(1.013,1.042)	1.334(1.276,1.395)	1.118
	>60	57(58.8%)	1.039(1.007,1.073)	1.937(1.866,2.010)	1.293
Division 7	0–14	79(40.7%)	1.034(1.002,1.066)	5.911(4.935,7.081)	1.296
	>60	92(47.4%)	1.026(1.011,1.041)	3.406(2.947,3.937)	1.324
Division 8	0–14	11(10.9%)	1.017(1.006,1.029)	1.373(1.264,1.493)	1.141
	>60	47(46.5%)	1.047(1.027,1.067)	2.750(2.685,2.817)	1.358
Division 9	0–14	30(43.5%)	1.025(1.017,1.033)	2.611(2.462,2.770)	1.175
	>60	24(34.8%)	1.017(1.006,1.028)	1.903(1.779,2.035)	1.187

CI: confident interval;

Percentage was derived by the number of geographic units for each age level divided by the total number of counties at each geographic division.

**Figure 4:**
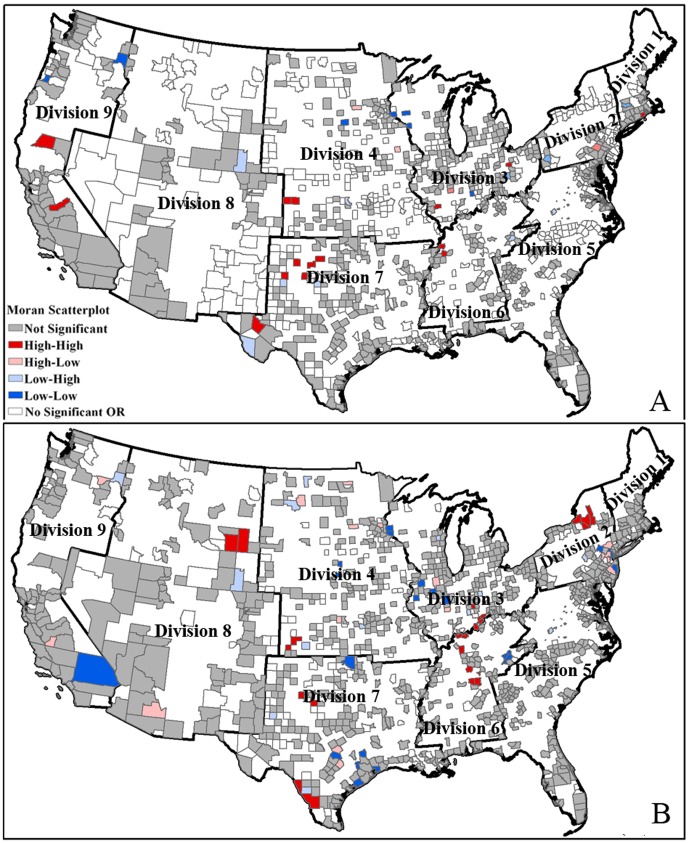
Clusters for benzene pollution exposure by age at county level. A local autocorrelation method is used to identify statistically significant hot spots, or cluster areas. High-High areas indicate high values near high values; Low-Low areas indicate low values near low values; High-Low areas indicate high values near low values; Low-High areas indicate low values near high values. (A) age (<14); (B) age (>60). Division 1 is New England; Division 2 is Mid-Atlantic; Division 3 is East North Central; Division 4 is West North Central; Division 5 is South Atlantic; Division 6 is East South Central; Division 7 is West South Central; Division 8 is Mountain; Division 9 is Pacific.

Figure 4 delineates the county level spatial clusters of benzene pollution exposure inequity based on data from Table 3. From Figure 4, the high-risk areas for the age group of 0–14 are located in Divisions 1, 3, 4, 6, 7, and 9, which includes the number of spatial cluster county units of 1, 2, 2, 2, 6, 2 respectively (Fig. 4A). Figure 4B shows high-risk areas for people age 60 years and over. It can be seen that these clusters were mainly located in Divisions 2–4 and 6–8, which includes the number of spatial cluster county units, which was 4, 7, 2, 7, 4, 2 respectively.

### Spatial Clustering of Air Pollution Exposure Inequity by Race

Table 4 delineates frequency of *OR*s greater than 1 by race characteristic at the county level in the United States and the nine divisions. It can be seen that racial minorities such as Blacks, American Indians, and Asians were exposed to higher levels of benzene pollution in some counties. For Blacks, Division 1 had the highest proportion (86.2%), followed by Division 3 (77.6%) and Division 2 (75.3%). The smallest proportion for Blacks was found in Division 8 (54.5%). For American Indians, Division 1 had the highest proportion (65.5%), followed by Division 9 (56.5%) and Division 3 (51.7%). The smallest proportion for American Indians was found in Division 6 and Division 7 (30.9%). For Asians, Division 1 had the highest proportion (82.8%), followed by Division 2 (77.8%) and Division 6 (70.1%). The smallest proportion for Asians was found in Division 7 (44.3%). For other races, Division 1 showed the highest level of exposure with (86.2%) followed by Divisions 9 and 3 (82.6%; 81.0%). The lowest exposure in this racial group was in Division 5 (61.7%). It could also be observed that the proportion of counties exposed to higher levels of benzene pollution by divisions is mostly more than 50% for the United States and the nine divisions, except for the American Indians in Divisions 2, 4, 5, 6, 7, and 8 and Asians in Division 7.

**Table 4:** Frequency of *OR*s greater than 1 by race at the county level in the United States and the nine divisions.

	Race	Count (percentage)	Minimum (95%CI)	Maximum (95%CI)	Mean
United States	Black	795(69.4%)	1.041(1.021,1.061)	56.589(48.884,65.510)	3.714
	American Indian	485(42.3%)	1.040(1.006,1.074)	14.726(10.112,21.445)	1.965
	Asian	679(59.2%)	1.044(1.016,1.072)	246.341(89.734,676.264)	3.157
	Other races	815(71.1%)	1.017(1.009,1.025)	11.887(8.812,16.034)	2.058
Division 1	Black	25(86.2%)	1.117(1.101,1.133)	9.809(9.470,10.160)	2.866
	American Indian	19(65.5%)	1.105(1.020,1.197)	3.161(2.945,3.394)	1.788
	Asian	24(82.8%)	1.220(1.185,1.257)	3.444(2.964,4.002)	2.038
	Other races	25(86.2%)	1.169(1.064,1.285)	6.110(5.941,6.284)	2.511
Division 2	Black	61(75.3%)	1.101(1.063,1.140)	17.066(16.205,17.973)	3.437
	American Indian	40(49.4%)	1.140(1.014,1.281)	2.788(2.497,3.114)	1.827
	Asian	63(77.8%)	1.044(1.016,1.072)	6.845(6.200,7.558)	2.009
	Other races	64(79.0%)	1.017(1.009,1.025)	7.097(6.915,7.283)	2.502
Division 3	Black	135(77.6%)	1.071(1.051,1.092)	37.255(26.109,53.160)	4.675
	American Indian	90(51.7%)	1.040(1.006,1.074)	3.824(1.769,8.269)	1.785
	Asian	94(54.0%)	1.049(1.014,1.086)	12.118(5.956,24.652)	2.891
	Other races	141(81.0%)	1.053(1.002,1.106)	6.173(4.613,8.261)	2.064
Division 4	Black	128(68.4%)	1.139(1.075,1.207)	50.856(7.124,363.056)	4.898
	American Indian	90(48.1%)	1.140(1.037,1.254)	14.726(10.112,21.445)	2.460
	Asian	110(58.8%)	1.075(1.023,1.132)	42.609(13.700,132.520)	4.063
	Other races	136(72.7%)	1.101(1.020,1.189)	11.886(9.798,14.419)	2.355
Division 5	Black	142(66.4%)	1.059(1.048,1.069)	56.589(48.884,65.510)	2.762
	American Indian	76(35.5%)	1.120(1.014,1.238)	14.161(8.893,22.550)	1.990
	Asian	130(60.7%)	1.050(1.035,1.066)	9.057(4.871,16.841)	2.061
	Other races	132(61.7%)	1.086(1.051,1.123)	7.285(6.750,7.862)	1.901
Division 6	Black	68(70.1%)	1.058(1.029,1.088)	16.902(13.287,21.502	4.093
	American Indian	30(30.9%)	1.117(1.005,1.241)	13.214(6.956,25.100)	2.254
	Asian	68(70.1%)	1.201(1.067,1.353)	246.341(89.734,676.264)	6.483
	Other races	67(69.1%)	1.157(1.021,1.312)	11.887(8.812,16.034)	2.023
Division 7	Black	130(67.0%)	1.060(1.020,1.102)	37.270(5.194,267.413)	3.631
	American Indian	60(30.9%)	1.096(1.012,1.188)	8.159(1.089,61.142)	1.817
	Asian	86(44.3%)	1.105(1.010,1.210)	20.688(2.850,150.144)	3.389
	Other races	126(64.9%)	1.041(1.010,1.073)	5.782(3.813,8.767)	1.939
Division 8	Black	55(54.5%)	1.127(1.098,1.157)	10.894(5.371,22.096)	2.691
	American Indian	41(40.6%)	1.200(1.101,1.308)	4.209(2.995,5.913)	1.892
	Asian	57 (56.4%)	1.066(1.023,1.111)	24.251(7.696,76.415)	2.845
	Other races	67(66.3%)	1.060(1.022,1.101)	4.781(4.079,5.605)	1.768
Division 9	Black	51(73.9%)	1.041(1.021,1.061)	13.205(8.974,19.430)	2.399
	American Indian	39(56.5%)	1.062(1.033,1.092)	2.192(2.131,2.255)	1.494
	Asian	47(68.1%)	1.064(1.043,1.086)	8.475(7.168,10.021)	1.848
	Other races	57(82.6%)	1.039(1.020,1.059)	3.091(3.049,3.133)	1.649

CI: confident interval;

Percentage was derived by the number of geographic units for each race level divided by the total number of counties at each geographic division.

**Figure 5:**
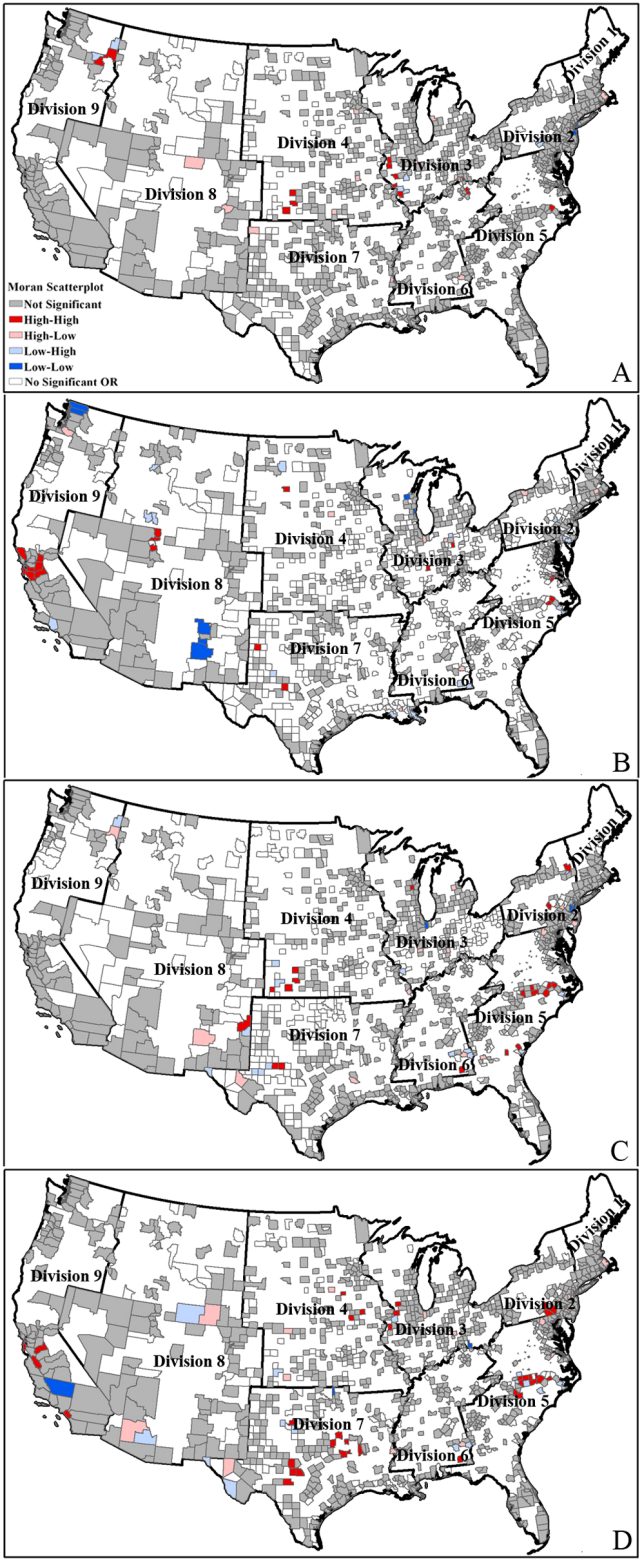
Clusters for benzene pollution exposure by race at county level. (A): Black; (B): American Indian; (C): Asian; (D): Other races. Division 1 is New England; Division 2 is Mid-Atlantic; Division 3 is East North Central; Division 4 is West North Central; Division 5 is South Atlantic; Division 6 is East South Central; Division 7 is West South Central; Division 8 is Mountain; Division 9 is Pacific.

Figure 5 shows the county level clusters of benzene pollution exposure inequity based on the results from Table 5. High-risk areas for Blacks were found in Divisions 3, 4, 5, 6, 9, which included the number of spatial cluster county units of 5, 3, 1, 3, 2 respectively (Fig. 5A). Figure 5B shows the high-risk clusters for American Indians. These cluster areas are mainly located in Divisions 3, 4, 5, 7, 8, 9, which included the number of spatial cluster county units is 2, 1, 6, 2, 3, 7 respectively. High-risk spatial cluster areas for Asians are located in Divisions 2, 4, 5, 6, 7, 8, with spatial cluster county units of 2, 4, 8, 1, 2, and 1 (Fig. 5C). High-risk spatial cluster areas of other races are located in Divisions 2, 3, 4, 5, 6, 7, 9, which included the spatial cluster county units of 3, 3, 3, 10, 1, 11, and 4 respectively(Fig. 5D).

**Table 5:** Frequency of *OR*s greater than 1 by educational attainment at the county level in the United States and the nine divisions.

	Educational attainment	Count (percentage)	Minimum (95%CI)	Maximum (95%CI)	Mean
United States	0–4	660(57.6%)	1.075(1.021,1.132)	26.923(6.594,109.924)	2.427
	5–8	586(51.1%)	1.045(1.001,1.092)	21.318(13.042,34.847)	1.757
	9–12	625(54.5%)	1.023(1.002,1.045)	9.982(8.204,12.146)	1.515
Division 1	0–4	19(65.5%)	1.272(1.099,1.472)	4.294(3.690,4.997)	2.355
	5–8	22(75.9%)	1.118(1.025,1.220)	2.606(2.422,2.804)	1.757
	9–12	21(72.4%)	1.051(1.014,1.089)	2.052(2.002,2.103)	1.510
Division 2	0–4	61(75.3%)	1.081(1.003,1.166)	4.946(4.790,5.108)	1.906
	5–8	51(63.0%)	1.059(1.014,1.105)	2.958(2.890,3.028)	1.566
	9–12	51(63.0%)	1.064(1.019,1.110)	2.303(2.257,2.350)	1.479
Division 3	0–4	109(62.6%)	1.167(1.052,1.296)	7.555(5.171,11.039)	2.310
	5–8	96(55.2%)	1.092(1.003,1.190)	3.030(2.706,3.394)	1.566
	9–12	108(62.1%)	1.041(1.001,1.083)	3.182(3.087,3.280)	1.450
Division 4	0–4	99(52.9%)	1.152(1.008,1.317)	17.858(12.621,25.268)	3.309
	5–8	80(42.8%)	1.106(1.021,1.198)	3.308(2.929,3.736)	1.616
	9–12	91(48.7%)	1.046(1.023,1.069)	2.685(2.518,2.864)	1.469
Division 5	0–4	117(54.7%)	1.144(1.042,1.255)	11.495(9.706,13.615)	2.120
	5–8	112(52.3%)	1.075(1.008,1.146)	21.318(13.042,34.847)	1.905
	9–12	120(56.1%)	1.023(1.002,1.045)	9.982(8.204,12.146)	1.590
Division 6	0–4	43(44.3%)	1.093(1.029,1.161)	3.184(2.804,3.616)	1.765
	5–8	34(35.1%)	1.112(1.007,1.227)	3.076(2.846,3.323)	1.590
	9–12	44(45.4%)	1.064(1.004,1.128)	2.083(1.961,2.213)	1.381
Division 7	0–4	110(56.7%)	1.143(1.004,1.301)	7.281(3.523,15.051)	2.295
	5–8	96(49.5%)	1.076(1.001,1.156)	5.169(3.627,7.367)	1.931
	9–12	102(52.6%)	1.042(1.010,1.074)	5.187(3.816,7.050)	1.586
Division 8	0–4	56(55.4%)	1.246(1.132,1.371)	26.923(6.594,109.924)	3.333
	5–8	48(47.5%)	1.291(1.184,1.408)	3.568(3.492,3.645)	2.038
	9–12	49(48.5%)	1.084(1.030,1.140)	2.350(2.049,2.697)	1.604
Division 9	0–4	46(66.7%)	1.075(1.021,1.132)	5.470(4.389,6.816)	2.141
	5–8	47(68.1%)	1.045(1.001,1.092)	3.220(3.179,3.262)	1.723
	9–12	39(56.5%)	1.068(1.025,1.112)	2.504(2.479,2.529)	1.472

CI: confident interval;

Percentage was derived by the number of geographic units for each education level divided by the total number of counties at each geographic division.

### Spatial Cluster of Air Pollution Exposure Inequity by Education

Table 5 delineates frequency of *OR*s greater than 1 by education characteristic at the county level in the United States and the nine divisions. Results indicate that individuals with less than 12 years education were exposed to higher levels of benzene pollution in some counties of the United States. For those with less than 4 years education, Division 2 had the highest proportion (75.3%), followed by Division 9 (66.7%) and Division 1 (65.5%). The smallest proportion for this same education group was found in Division 6 (44.3%). For the education level of 5 to 8 years, Division 1 had the highest proportion (75.9%), followed by Division 9 (68.1%) and Division 2 (63.0%). The smallest proportion for this education group was found in Division 6 (35.1%). For the education level of 9 to 12 years, Division 1 bore the greatest exposure with (72.4%) followed by Divisions 2 (63.0%) and 3 (62.1%). The lowest exposure for this age group was in Division 6 (45.4%). We also observed that the proportion of the total number of counties exposed to high levels of benzene pollution by divisions was more than 50% for the United States and the nine divisions, except for the education levels of 5 to 8 years in Divisions 4, 6, 7, 8 and the education levels ranging from 9 to 12 years in Divisions 4, 6, 8.

**Figure 6:**
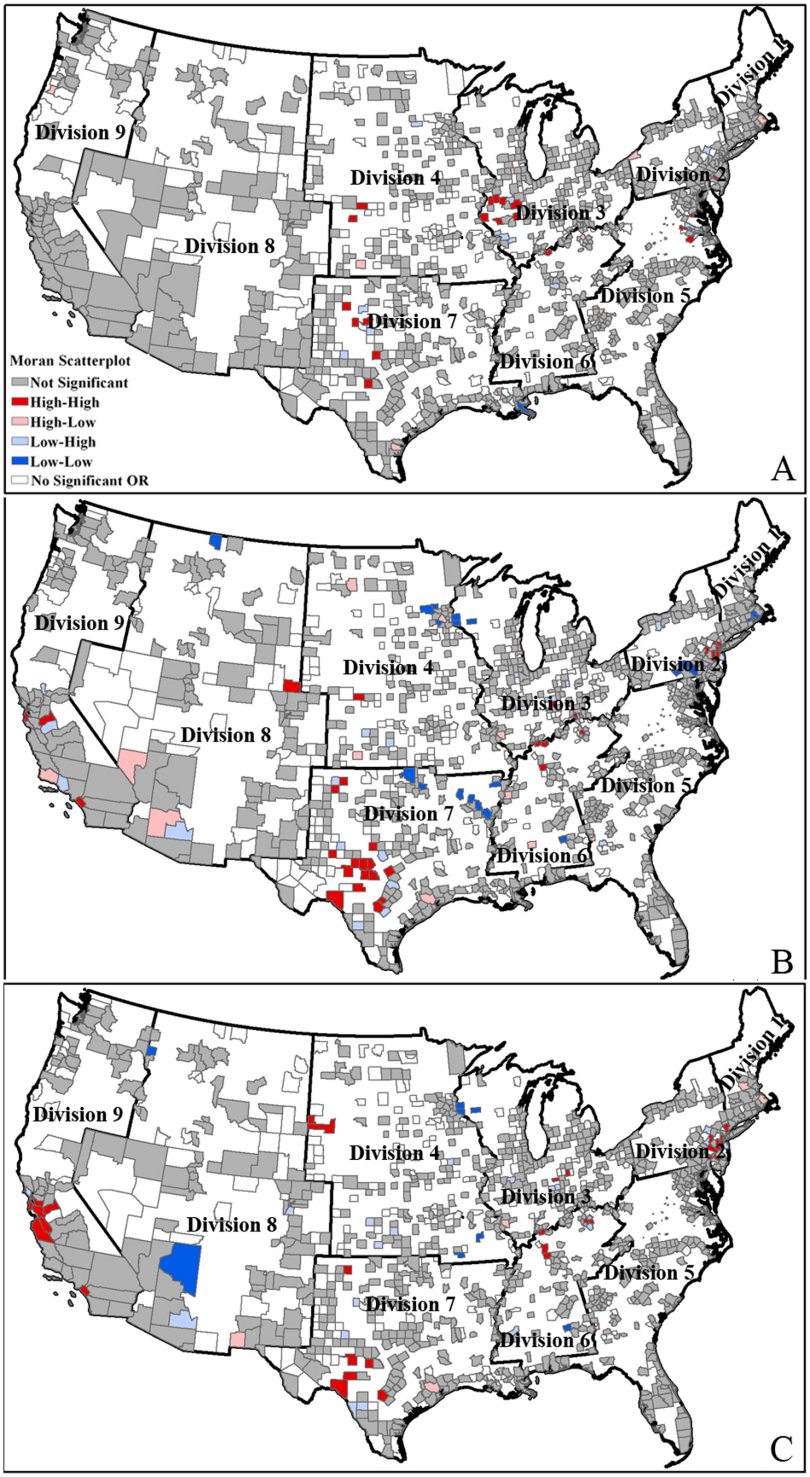
Clusters for benzene pollution exposure by education at county level. (A): Educational attainment (0–4); (B): Educational attainment (5–8); (C): Educational attainment (9–12). Division 1 is New England; Division 2 is Mid-Atlantic; Division 3 is East North Central; Division 4 is West North Central; Division 5 is South Atlantic; Division 6 is East South Central; Division 7 is West South Central; Division 8 is Mountain; Division 9 is Pacific.

Figure 6 shows the county levels inequality of benzene pollution exposure based on information in Table 5. High-risk areas for education level less than 4 years were located in Divisions 2, 3, 4, 5, 6, 7, which included the number of spatial cluster county units of 1, 7, 2, 6, 1, 5, respectively (Fig. 6A). Figure 6B shows that high-risk areas for people of the 5–8 years of education level were mainly located in Divisions 2, 3, 4, 6, 7, 8, 9, which are associated with spatial cluster county units of 8, 1, 1, 5, 15, 1, 3 respectively. High-risk areas for education level between 9 and 12 years were located in Divisions 2, 3, 4, 6, 7, 9, which included the number of spatial cluster county units of 10, 2, 2, 5, 6, 7 respectively (Fig. 6C).

### Spatial Cluster of Air Pollution Exposure Inequity by Income

Table 6 delineates frequencies of *OR*s greater than 1 by income characteristics at the county level in the United States and by the nine US Census Bureau divisions. From Table 6, it can be seen that people belonging to low income groups were exposed to higher levels of benzene pollution in some counties across the United States. For the low-income group, Division 1 had the highest proportion of residents with high exposure (65.5%), followed by Division 2 (60.5%) and Division 3(42.5%). The smallest proportion for the low-income group was found in Division 7 (10.3%). We also observed that the proportion of counties exposed to higher levels of benzene pollution by Division is mostly less than 50% for the United States and the nine divisions, except for the low-income group in Divisions 1 and 2.

**Table 6:** Frequency of *OR*s greater than 1 by income at the county level in the United States and the nine divisions.

	Income	Count (percentage)	Minimum (95%CI)	Maximum (95%CI)	Mean
United States	Low income	346(30.2%)	1.029(1.006, 1.053)	9.809(9.558,10.067)	3.340
Division 1	Low income	19(65.5%)	1.149(1.136,1.163)	9.809(9.558,10.067)	4.116
Division 2	Low income	49(60.5%)	1.040(1.018,1.062)	9.653(9.380,9.935)	3.263
Division 3	Low income	74(42.5%)	1.064(1.041,1.088)	9.497(9.300,9.699)	3.673
Division 4	Low income	47(25.1%)	1.057(1.041,1.073)	9.095(8.680,9.530)	3.437
Division 5	Low income	58(27.1%)	1.056(1.046,1.065)	9.269(9.026,9.520)	9.269
Division 6	Low income	31(32.0%)	1.029(1.006,1.053)	9.436(9.316,9.558)	3.039
Division 7	Low income	20(10.3%)	1.107(1.088,1.127)	8.754(8.393,9.130)	2.642
Division 8	Low income	20(19.8%)	1.087(1.074,1.101)	9.175(9.126,9.224)	3.936
Division 9	Low income	28(40.6%)	1.178(1.150,1.208)	8.609(8.458,8.764)	3.079

CI: confident interval;

Percentage was derived by the number of geographic units for low income level divided by the total number of counties at each geographic division.

**Figure 7:**
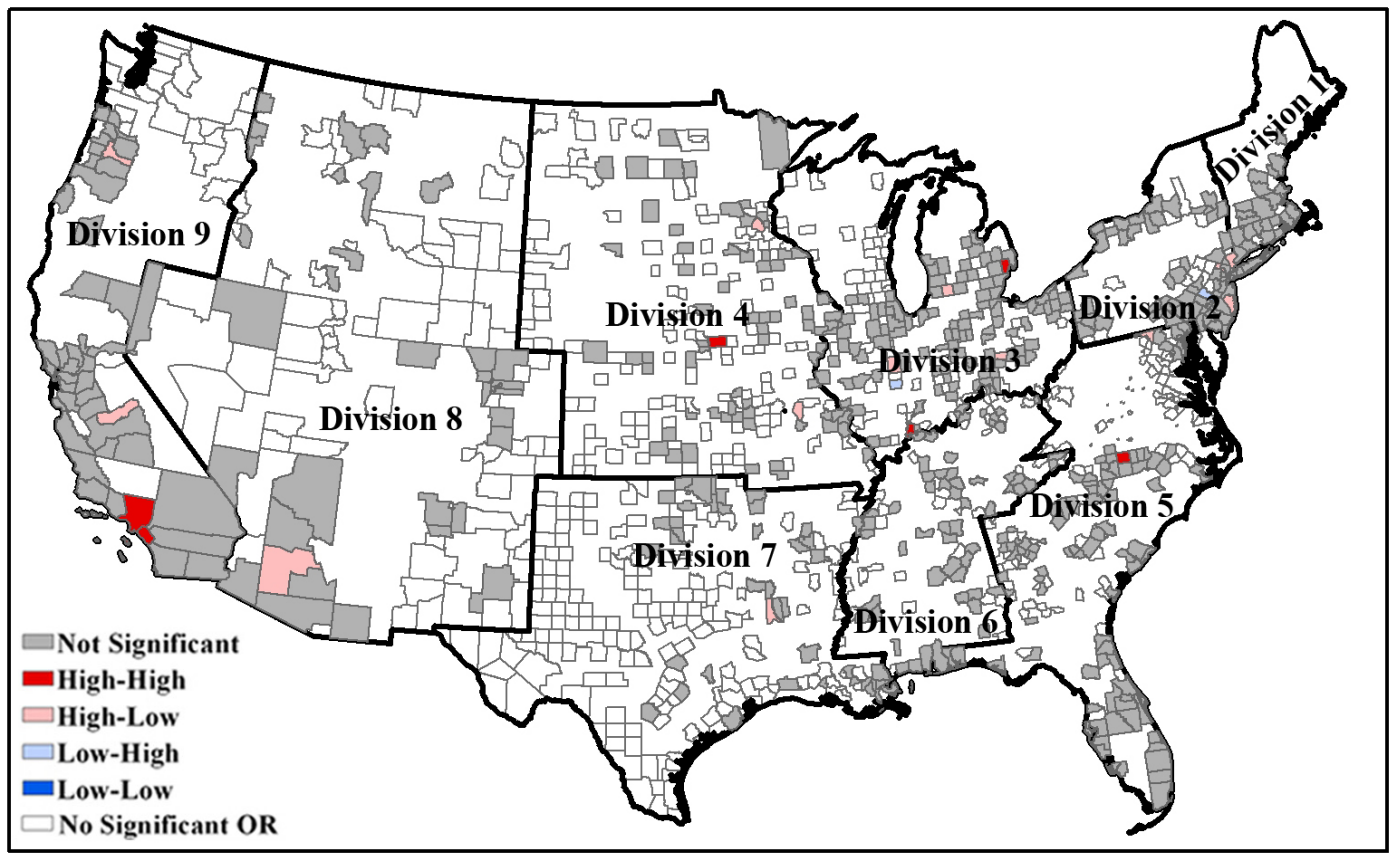
Clusters for benzene pollution exposure by income at county level. Fig. 7 shows High-High areas are high values cluster areas, in which people with low income exposed to higher level of benzene pollution than those with high income. Division 1 is New England; Division 2 is Mid-Atlantic; Division 3 is East North Central; Division 4 is West North Central; Division 5 is South Atlantic; Division 6 is East South Central; Division 7 is West South Central; Division 8 is Mountain; Division 9 is Pacific.

Figure 7 details the county level spatial clusters of benzene pollution exposure inequity based on data from Table 6. As shown in Figure 7, the high-risk areas for the low-income groups are located in Divisions 3, 4, 5 and 9, which include the number of spatial cluster county units of 2, 1, 1, 2 respectively.

## Discussion

This study is among the first spatial assessments of the inequities of air pollution exposure across the entire continental United States at the census tract scale. The results demonstrated that disparities in benzene air pollution exposure could help explain health disparities by age, race, educational attainment, and income. Although there has been a national decrease in health disparities between 1990 and 1998 [41], some divisions have reported an increase in disparities during the same period [42]. Marshall [34] found environmental inequities of air pollution exposure in California's South Coast Air Basin, which persisted even after accounting for covariates such as population density, travel distance, mean differences between whites and nonwhites were 16–40% among the five pollutants.

A unique insight of this current study is that it highlighted spatial clusters of air pollution exposure inequity by race. Previous studies have shown that hazardous waste and industrial facilities were commonly located in or close to communities with populations that are of disproportionately higher proportions of minority or low-income individuals [43]. Our study extends the findings of previous studies by incorporating the spatial perspective of these inequities.

Minority neighborhoods tend to have higher rates of mortality, morbidity, and are more likely to be influenced by health risk factors than white neighborhoods, even after accounting for economic and other characteristics [44]. According to Gee and Takeuchi [45], differential residential locations come with differential levels of exposure to health risks. In particular, neighborhood stressors and pollution sources are related to adverse health conditions, which are counterbalanced by neighborhood resources. When community stressors and pollution sources outweigh neighborhood resources, levels of community stress manifest or increase. Community stress is a state of ecological vulnerability that may translate into individual stressors, which in turn may lead to individual stress. Individual stress may then make individuals more vulnerable to illness when they are exposed to environmental hazards. Furthermore, compromises in individual and community health may further weaken community resources, leading to a vicious cycle [46].

In addition, a key finding in our study is the significant inequities of air pollution exposure by educational attainment and income in the United States. For educational attainment based inequities, the results followed those of a previous study of 20 US cities which revealed strong (although not statistically significant) associations between PM_10_ and mortality for less educated subjects [47] as well as a study from Shanghai, China that showed an association between lower education and greater impact of air pollution-attributed mortality [48]. As to income attainment-based inequities, although the income in most census tracts across United States in this study exceeds the national poverty guideline for the same period, significant and large *OR*s were observed for counties with relatively low income. This would indicate that people belonging to low income groups were more likely to be exposed to higher levels of benzene pollution in the United States relative to their higher income counterparts.

It should be noted that as this study is fairly unique in the methodology employed (e.g. spatial autocorrelation) for investigating environmental and socio-demographic inequities (geographic unit, methods of statistical analysis, exposure assessment procedures and definition of deprivation), our results are difficult to compare to other studies in relativistic terms. As more studies using this type of methodology are performed, a more comprehensive comparison will be possible. However, the results provided in this study would be highly applicable in other areas of research such as causal analysis of disease clusters, environmental policy targeting, and human rights policy making over large geographical areas.

Similar to previous analyses, the results of this study must be interpreted with caution. For example, since this study only examined a single type of air pollutant (i.e. benzene), our findings may not be generalizable to the cumulative effect of all other types of air pollutants. Further, our racial disparity analysis was only restricted to the classification of Blacks, American Indians, Asians and “Other races”. Thus, we do not know if the interactive relationships uncovered here would hold true for Pacific Islanders who were probably combined with Asians or whether the results would change, which might make it be reasonable to identify Pacific Islanders as ‘Other race’ in the categorization. This study may also mask rural/urban characteristics when analyzing racial inequities in air pollution exposure. Similar to other ecological studies, this paper used aggregate data (e.g. census tract level) and could not incorporate individual-level information such as individual migration, time length of residence, and location exposure differences between work, recreation and living. Finally, as this study does not test any causal hypotheses, we could not explain how or why race, age, educational attainment and income interact to produce air pollution inequity.

Another limitation of our data source is that, in Canada and the United States, census tracts are often referred to as a representation of the neighborhood [49]. However, it has been demonstrated that these census units do not represent underlying social boundaries and may depict the artifacts of administrative rules of a putative system [50]. Hence, it is sometimes difficult to tease out if the results of the analysis are representative of the reality or if they are the results of using a certain type of geographical unit [51].

To remedy the limitations of current studies, this paper identifies a set of overarching recommendations. Based on our results, scientists and community leaders should work in partnership to prioritize research needs, gather data, assess other air pollutants beyond benzene, and test interventions that will influence public policy in order to protect the health of all, including those living in communities of color and places that are economically deprived. Policy-makers can also enhance existing services that assist vulnerable groups and/or susceptible individuals to help close the disparity of exposure.

## Conclusions

In summary, this study revealed that there are disproportionate exposures to benzene air pollution by a range of factors including age, race, education attainment and income in the United States. Spatial autocorrelation was also shown to be a valuable tool in this study to analyze how socio-demographic variables can influence the spatial patterns of air pollution exposure. However, further work is needed to inform policy-makers so that they can respond to the challenges and expectations that will improve environmental conditions for all underrepresented groups in the United States and beyond.
